# Repetitive Mild Traumatic Brain Injury Alters Glymphatic Clearance Rates in Limbic Structures of Adolescent Female Rats

**DOI:** 10.1038/s41598-020-63022-7

**Published:** 2020-04-10

**Authors:** Jennaya Christensen, David K. Wright, Glenn R. Yamakawa, Sandy R. Shultz, Richelle Mychasiuk

**Affiliations:** 1 Department of Psychology, University of Calgary, Calgary, Alberta Canada; 2 Department of Neuroscience, Central Clinical School, Monash University, Melbourne, Victoria Australia; 3 Department of Medicine, University of Melbourne, Parkville, Victoria Australia; 4 Hotchkiss Brain Institute, University of Calgary, Calgary, Alberta Canada; 5 Alberta Children’s Hospital Research Institute, University of Calgary, Calgary, Alberta Canada

**Keywords:** Neuroscience, Neurological disorders

## Abstract

The glymphatic system is the macroscopic waste clearance system for the central nervous system. Glymphatic dysfunction has been linked to several neurological conditions, including traumatic brain injury (TBI). Adolescents are at particularly high risk for experiencing a TBI, particularly mild TBI (mTBI) and repetitive mTBI (RmTBI); however, glymphatic clearance, and how it relates to behavioral outcomes, has not been investigated in this context. Therefore, this study examined glymphatic function in the adolescent brain following RmTBI. Female adolescent Sprague Dawley rats were subjected to either three mTBIs or sham injuries spaced three days apart. One-day after their final injury, the animals underwent a beam walking task to assess sensorimotor function, and contrast-enhanced MRI to visualize glymphatic clearance rate. Behavioural measures indicated that the RmTBI group displayed an increase in loss of consciousness as well as motor coordination and balance deficits consistent with our previous studies. The contrast-enhanced MRI results indicated that the female adolescent glymphatic system responds to RmTBI in a region-specific manner, wherein an increased influx but reduced efflux was observed throughout limbic structures (hypothalamus, hippocampus, and amygdala) and the olfactory bulb but neither the influx or efflux were altered in the cortical structures (primary motor cortex, insular cortex, and dorsolateral prefrontal cortex) examined. This may indicate a role for an impaired and/or inefficient glymphatic system in the limbic structures and cortical structures, respectively, in the development of post-concussive symptomology during adolescence.

## Introduction

In contrast to the periphery, the lymphatic vasculature, which actively removes metabolic waste and excess interstitial fluid (ISF), is absent from the central nervous system (CNS)^1^. Instead, the glymphatic system, named for its dependence on glial water flux and functional resemblance to the lymphatic system, satisfies this requirement for the CNS^2^. The glymphatic system utilizes paravascular tunnel pathways to eliminate macroscopic waste, such as soluble proteins and metabolites, from the CNS^3^. Importantly, this highly organized system is responsible for the removal of neurotoxic compounds, including amyloid-β and tau^4^. Additionally, this waste removal system is also believed to assist in the brain-wide distribution of various compounds critical to neurological function, such as glucose, amino acids, neuromodulators, lipids, and growth factors^3^.

The glymphatic system relies on three specialized pathways; para-arterial cerebrospinal fluid (CSF), paravenous ISF, and the intracellular trans-astrocytic pathway^4^. These three systems are responsible for influx, efflux, and connectivity between CSF and ISF, respectively. More specifically, flow of CSF follows the paravascular pathways near arteries and interacts with the paravenous ISF to eliminate macroscopic waste^5^. The aquaporin-4 (AQP4) water channels found on astrocytic end feet are believed to be play an critical role in the exchange of CSF and ISF^5^. Ultimately, these interstitial proteins and solutes are degraded by the liver, which circumvents the requirement for local protein processing and degradation in the CNS^4^.

Children and adolescents represent two of the highest risk age groups for experiencing a traumatic brain injury (TBI), particularly mild TBI (mTBI) and repetitive mTBI (RmTBI)^6–8^. More specifically, previous research has indicated that 1 in 5 children will have sustained a mTBI by the age of 16^9^. Adolescents are at particularly high risk for experiencing persistent post-injury deficits, given the dramatic changes in neurological structure and organization that occurs during this critical stage of development^9–12^. Repeated head trauma is particularly damaging to the brain as the altered cerebral physiology from injuries increases the brain’s vulnerability to the effects of additional blows^13,14^. There are several cellular mechanisms responsible for this increase in susceptibility to repeated trauma, including metabolic dysfunction and amplified axonal damage^13,15–17^. Furthermore, evidence suggests that intra-axonal tau accumulation, tau phosphorylation, amyloid-β buildup, and plaque formation are consequences of RmTBI that correlate with injury severity and promote neurodegeneration^13,18–22^. Correspondingly, an animal model of moderate TBI has been shown to impair glymphatic clearance function and incite accumulation of phosphorylated tau^5^, suggesting that glymphatic dysfunction plays a key role in the development of TBI-related neuropathology and neurological deficits.

Therefore, this study aimed to examine glymphatic function in the female adolescent brain following RmTBI via utilization of contrast-enhanced *in vivo* MRI. We hypothesized that RmTBI would impair the glymphatic system, producing reductions in the glymphatic clearance rate of RmTBI animals in comparison to controls.

## Methods

### Animals

This experiment was approved by the University of Calgary Conjoint Faculties Research Ethics board and was carried out in accordance with the Canadian Council of Animal Care. Female adolescent Sprague Dawley rats were utilized in this study. All animals were kept in an animal housing facility throughout the course of the experiment. The animal housing facility maintained a 12:12-h light:dark cycle and was temperature controlled (21 °C). All animals were given *ad libitum* access to water and standard laboratory chow throughout the duration of this study. Once the rats were weaned at postnatal day (P) 21, they were housed in cages of 4.

### RmTBI

Within each cage, two of the rats were randomly assigned to the RmTBI group while the remaining two were assigned to the sham group. The rats assigned to the RmTBI group received three mTBI’s, which occurred on P30, P34, and P38. The last mTBI occurred one day before the MRI scanning date.

Prior to both the mTBI and sham injuries, the rats underwent brief administration (~60 s) of isoflurane anesthetic via an induction chamber. An adequate loss of consciousness was determined by a lack of response to a toe-pinch. The mTBI injuries were administered using a lateral impact device, which utilized a 50 g weight propelled at an average speed of 9.02 ± 0.18 m/s. After anesthetization, the rats in the RmTBI group were placed in a prone position with the left temporal lobe area rested against a protective headguard situated directly opposite the impactor. The weight was propelled toward this side of the rat’s head using pneumatic pressure and upon impact, spun the rat through a horizontal 180° rotation. No lacerations to the eye or head resulted from this injury. The rats in the sham injury group were placed in the same position within the lateral impact device as the RmTBI group but were removed without injury. After both the mTBI and sham injury, the rats received topical administration of lidocaine (AstraZeneca, Mississauga, ON) to the left temporal lobe area before being transferred to a clean heated cage for recovery.

### Behavioural testing

Two specific behavioural tests (time-to-right and beamwalking) were chosen to verify that both the RmTBI and sham injury groups exhibited behavioural outcomes consistent with our previous studies^23–26^. Time-to-right for both injury groups was measured immediately following the injuries to assess loss of consciousness. This measurement consisted of the time between injury impact and when the animal regained muscle tone. One day after the third mTBI or sham injury, the animals underwent the beam walking task, which assessed balance and motor coordination capabilities^27^. This task requires the rats to walk across a tapered beam situated 1 m above the ground to reach their home cage. Hindleg footslips were recorded for each of the 4 testing trials.

### Gadovist injection for visualization of glymphatic clearance

First, the rat was weighed and injected with 100 mg/kg of ketamine xylazine (9:1 ketamine to xylazine volume ratio). Once the rat had lost consciousness, which was determined by a lack of response to a toe-pinch, its head was shaved. Next, the rat was placed in a stereotaxic frame and the injection area was disinfected with Betadine (Purdue Pharma, Pickering, ON). The rat’s body temperature was monitored with an anal temperature probe and a pliable modified nose cone was fixed snugly around the rat’s head to administer oxygen and 1% isoflurane when required. The injector was made up of a fine 23G biopsy needle (Cook Medical, Bloomington, IN), polyethylene tubing, and a Hamilton syringe. The injector was placed within the stereotaxic arm and descended into the cisterna magna. The correct placement of the injector was confirmed by the ability to draw up CSF. Once the position of the cisterna magna was determined, 80 μl of 0.03 mM Gadovist (Bayer, Mississauga, ON) diluted in sterile saline was injected at a rate of ~1.6 μl/min. The rate of 1.6 uL/min was utilized based on previous studies that found this rate to not increase intracranial pressure^28^ and also less than or similar to the rat physiological CSF production rate^29,30^. When the injection was complete, the rat was removed from the stereotaxic frame and placed back in its cage for temporary recovery. The rat was allowed to regain consciousness for 15 minutes before being injected with a 50 mg/kg dose of ketamine xylazine (9:1 ketamine to xylazine volume ratio). The rat’s body temperature was then again monitored via a temperature probe from outside the MRI scanner until 2 hours and 15 minutes had passed from the start of injection.

### MRI procedures

For optimal glymphatic scanning, pilot testing by our laboratory determined that scanning should begin 2.5 hours after the start of injection. Therefore, at 2 hours and 15 minutes post-injection initiation, the rat was positioned within a 9.4 T Bruker MRI. The animal’s body temperature and breathing rate were monitored while in the scanner with anesthesia maintained using 1% isoflurane in 100% oxygen. A water vial was positioned next to the rat’s head for normalization of signal intensity. Once the rat had been correctly positioned within the MRI, an initial scan was performed to confirm successful injection of the Gadovist. If the injection was deemed successful, the experimental scanning was initiated, which occurred approximately 2½ hours after the start of injection. Whole brain T1-weighted images were acquired using the following imaging parameters: repetition time (TR) = 18 ms; echo time (TE) = 2.6 ms; flip angle (FA) = 15°; field of view (FOV) = 32 × 28 × 20 mm^3^; matrix = 160 × 140 × 100; and resolution = 0.2 × 0.2 × 0.2 mm^3^. Acquisitions were repeated every 5 minutes for a total duration of 3 hours.

### *In Vivo* MRI glymphatic clearance analysis

Prior to region of interest (ROI) tracing and analysis, MRI scans from each rat were evaluated for adequate Gadovist signal intensity. This evaluation rendered the exclusion of 3 rats (2 RmTBI and 1 sham) due to inadequate signal intensity, thus, making the sample sizes: RmTBI (n = 6) and sham (n = 5). Next, masks were created for the left and right hemispheres of the primary motor cortex, insular cortex, dorsolateral prefrontal cortex (DLPFC), hippocampus, amygdala, hypothalamus, and olfactory bulb on the MRI scans for each rat using the ITK-SNAP program. The location and quantity of the image slices and voxels for each mask were kept consistent in each set of rat MRI scans. Initial analysis demonstrated that there were no significant hemispheric differences, therefore the left and right masks for each ROIs were merged for statistical analysis.

Three outcome measures were generated for each brain region: maximum difference in signal intensity, amount of time from injection initiation required to reach maximum signal intensity, and the time between maximum and minimum signal intensity. A larger *maximum difference* indicates a higher *glymphatic clearance rate*, given that a larger maximum difference indicates that the Gadovist, which generates the signal intensity, is clearing from the paravascular pathways to a greater degree.

For the amount of time post-injection initiation required to reach *maximum signal intensity*, the less amount of time required to achieve maximum signal intensity, the higher the glymphatic clearance *influx* rate. This outcome measure provides an indication of how quickly the Gadovist infuses into the paravascular pathways of the glymphatic system throughout the brain. Since the rate of influx represents a component of the glymphatic system, it is linked to the clearance rate.

As for the time *between maximum and minimum signal intensity*, greater values for this measure indicate a reduced glymphatic clearance rate, since this signifies a slower clearance of the Gadovist from the paravascular pathways. Thus, this outcome measure provides an interpretation of glymphatic *efflux*.

### Statistical analysis

One-way ANOVAs with Injury (RmTBI or sham) as a factor were conducted for the behavioural measures and the MRI measures. All analyses were run using SPSS 24.0 for MAC and *p* values of <0.05 were considered significant. All graphs display means ± standard error of the mean (SEM). All data will be made available upon request to the corresponding author.

## Results

### Behavioural testing

**Figure 1:**
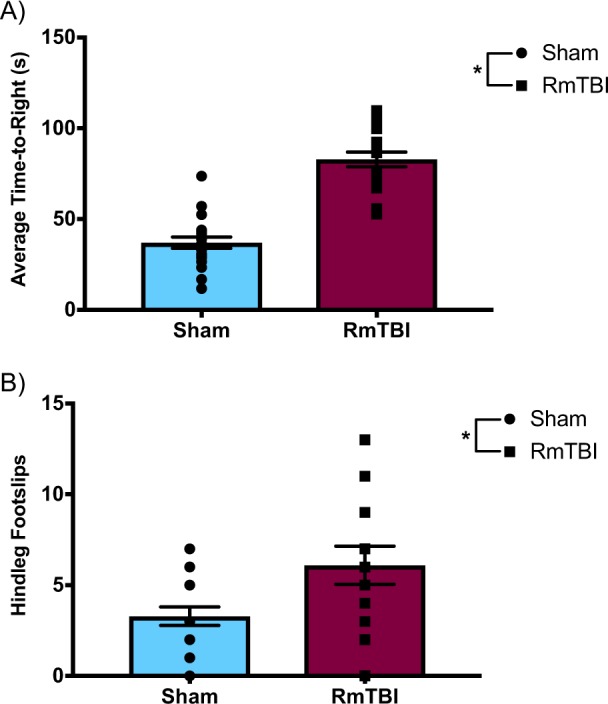
Scatter plot with bar graphs displaying the one-way ANOVA results for (**A**) average time-to-right and (**B**) hindleg footslips. Mean ± standard error; * main effect of injury, *p* < 0.05.

Results from the two behavioural tests chosen to verify the behavioural outcomes of the RmTBI and sham injury groups, were consistent with findings from our previous studies. The one-way ANOVA for time-to-right demonstrated a main effect of injury (*F*_(1, 36)_ = 78.183, *p* < 0.001) whereby the animals in the RmTBI group required significantly more time to right themselves following injury compared to the sham animals, demonstrating an increased loss of consciousness (Fig. 1A).

The one-way ANOVA for beam walking hindleg footslips exhibited a main effect of injury (*F*_(1, 36)_ = 4.976, *p* = 0.032), whereby RmTBI animals experienced significantly more hindleg footslips in comparison to the sham animals, suggesting impaired balance and motor coordination (Fig. 1B).

### Glymphatic clearance rate

**Figure 2:**
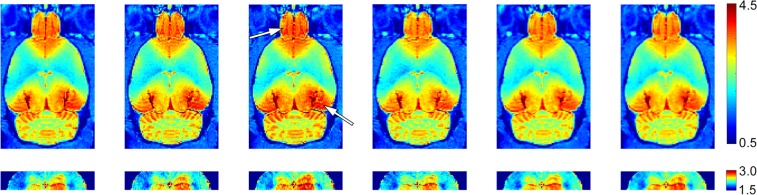
Sequential (left-to-right) T1-weighted images showing increasing Gadovist concentration in the olfactory bulb and hippocampus. The hippocampus is shown with increased contrast below. White arrows indicate regions of maximum signal intensity.

For each ROI, we generated 3 outcome measures: maximum difference in signal intensity, amount of time from injection initiation required to reach maximum signal intensity, and time between maximum and minimum signal intensity. There were no hemisphere effects in any of the ROI’s, thus, left and right hemispheres were combined for each brain region. Figure 2 provides an illustrative example of the change in Gadovist signal intensity over the 3-hour scan period.

**Figure 3:**
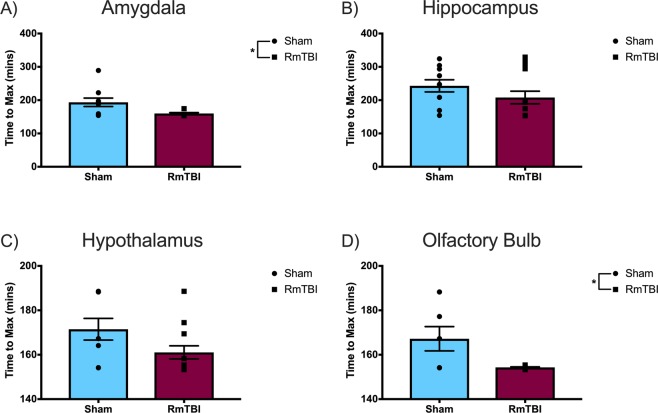
Scatter plot with bar graphs displaying the one-way ANOVA results for the time required to reach maximum signal intensity, a measure of glymphatic *influx*, for the (**A**) amygdala, (**B**) hippocampus, (**C**) hypothalamus, and (**D**) olfactory bulb. Time to max is calculated from the start of contrast agent injection. Means ± standard error; * main effect of injury, *p* < 0.05.

The time to reach maximum signal intensity was calculated as a measure of glymphatic *influx*, an important factor in glymphatic clearance (Fig. 3). With respect to this outcome measure, the results of the one-way ANOVA for the primary motor cortex (*F*_(1, 18)_ = 0.233, *p* = 0.635), insular cortex (*F*_(1, 18)_ = 0.278, *p* = 0.605), and hippocampus (*F*_(1, 18)_ = 1.666, *p* = 0.210) did not reveal any significant differences between the RmTBI and sham injury groups. Conversely, the one-way ANOVA for the olfactory bulb (*F*_(1, 18)_ = 10.662, *p* = 0.004) and amygdala (*F*_(1, 18)_ = 5.878, *p* = 0.026) demonstrated significant differences between the RmTBI and sham injury groups, wherein the RmTBI group required less time to reach maximum signal intensity, indicating an increase in glymphatic *influx* in comparison to sham animals.

**Figure 4:**
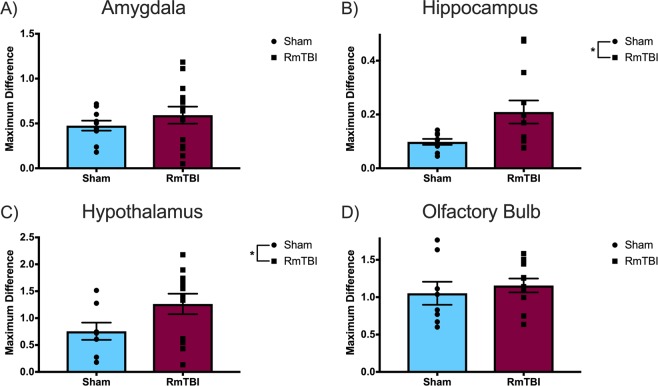
Scatter plot with bar graphs displaying the one-way ANOVA results for maximum difference in signal intensity, a measure of glymphatic *efflux*, for the (**A**) amygdala, (**B**) hippocampus, (**C**) hypothalamus, and (**D**) olfactory bulb. Mean ± standard error; * main effect of injury, *p* < 0.05.

The maximum difference in signal intensity was calculated as a measure of glymphatic *efflux* (Fig. 4). In regards to maximum difference in signal intensity, the one-way ANOVA for the primary motor cortex (*F*_(1, 18)_ = 0.167, *p* = 0.687), DLPFC (*F*_(1, 18)_ = 0.264, *p* = 0.613), insular cortex (*F*_(1, 18)_ = 0.001, *p* = 0.971), olfactory bulb (*F*_(1, 18)_ = 1.502, *p* = 0.236), and the amygdala (*F*_(1, 18)_ = 1.688, *p* = 0.210) did not reveal any significant effects of injury. On the other hand, the hypothalamus (*F*_(1, 18)_ = 6.058, *p* = 0.025) and the hippocampus (*F*_(1, 18)_ = 4.809, *p* = 0.041) exhibited significant differences between the RmTBI and sham injury groups, wherein the RmTBI group displayed a significantly greater maximum difference in signal intensity, which is indicative of an increased glymphatic clearance rate.

**Figure 5:**
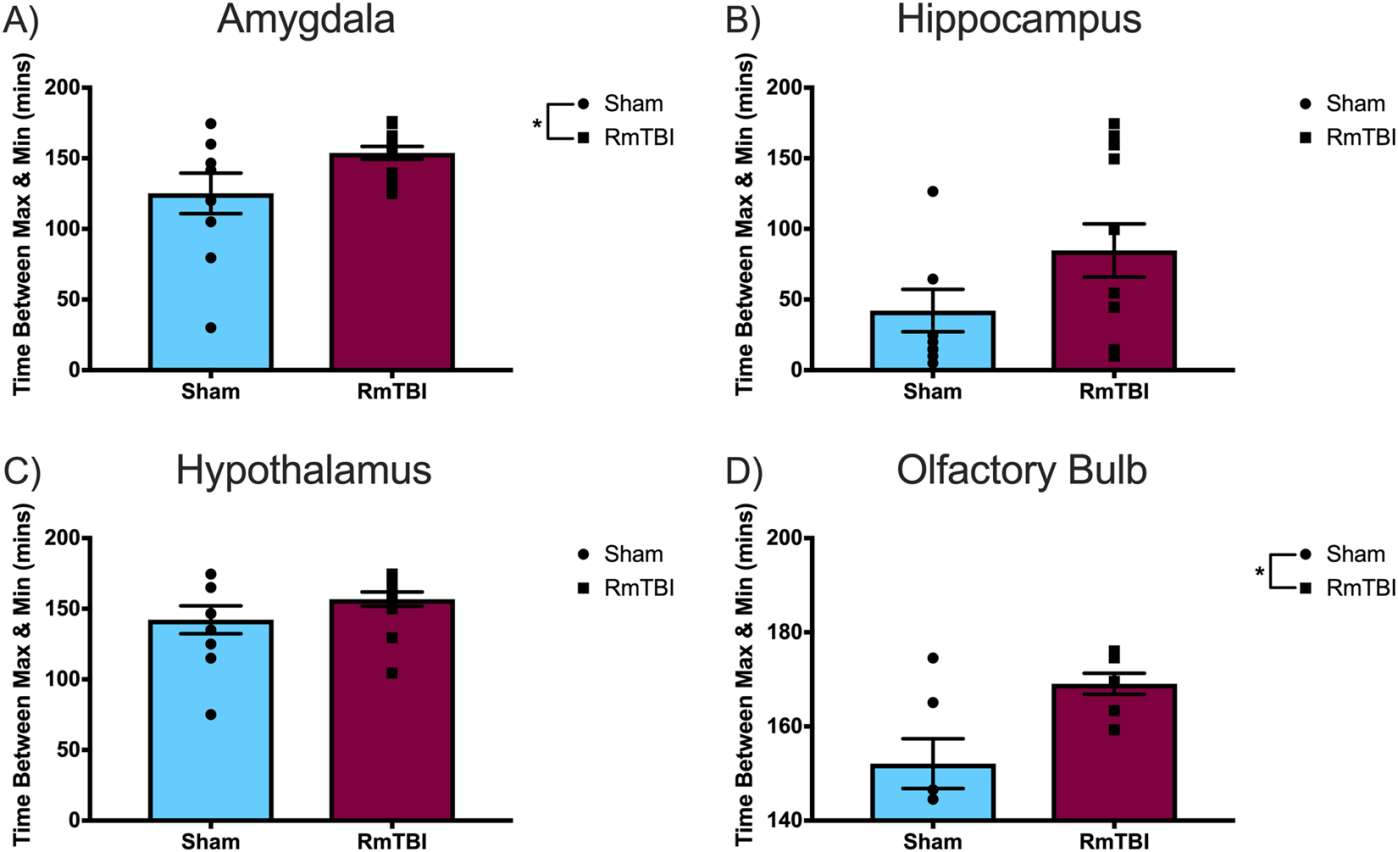
Scatter plot with bar graphs displaying the one-way ANOVA results for time between maximum and minimum signal intensity, a measure of glymphatic *efflux*, for the (**A**) amygdala, (**B**) hippocampus, (**C**) hypothalamus, and (**D**) olfactory bulb. Mean ± standard error; * main effect of injury, *p* < 0.05.

The time between maximum and minimum signal intensity was also calculated as a measure of glymphatic *efflux* but revealed different results than the aforementioned efflux outcome measure (Fig. 5). The one-way ANOVA for the primary motor cortex (*F*_(1, 18)_ = 2.067, *p* = 0.169), DLPFC (*F*_(1, 18)_ = 1.882, *p* = 0.184), insular cortex (*F*_(1, 18)_ = 0.087, *p* = 0.772), hippocampus (*F*_(1, 18)_ = 2.841, *p* = 0.107), and the hypothalamus (*F*_(1, 18)_ = 2.022, *p* = 0.169) did not display any significant effects. However, the amygdala (*F*_(1, 18)_ = 4.475, *p* = 0.047) and olfactory bulb (*F*_(1, 18)_ = 8.775, *p* = 0.008) demonstrated significant effects of injury, in which the RmTBI group exhibited a greater time between maximum and minimum signal intensity, which suggests a decreased glymphatic clearance in the RmTBI group.

## Discussion

Given that RmTBI is associated with neurodegenerative disorders in which tau and amyloid-β protein aggregates are pathological trademarks, it seems possible that RmTBI causes impairment of the glymphatic system, which may play a key role in the development of the behavioural and neurological deficits associated with RmTBI. The two behavioural tests we conducted identified deficits in the RmTBI group that were consistent with earlier studies. With regards to time-to-right, animals in the RmTBI group required significantly more time to regain consciousness compared to the sham animals, which is consistent with the literature^26,31,32^. The beam walking results indicated that RmTBI animals experienced significantly more hindleg footslips than sham animals, demonstrating the presence of balance and motor coordination deficits following RmTBI, which is also consistent with previous findings^26,31,32^.

We hypothesized that, similar to severe TBI^5^, RmTBI would impair glymphatic system function, resulting in reduced glymphatic clearance rates in the RmTBI animals compared to the controls. Interestingly, in the limbic structures (hypothalamus, hippocampus, and amygdala) and olfactory bulb, the RmTBI group displayed significant differences and trends towards significance indicative of increased glymphatic influx following RmTBI. However, the outcome measures indicative of glymphatic efflux demonstrated that the olfactory bulb and limbic structures of the RmTBI group are clearing waste more slowly than the sham injury group. Thus, as hypothesized, the RmTBI group displayed results that point towards impaired glymphatic function, specifically within the efflux element of the system. This disturbance in glymphatic function may contribute to post-concussive symptomology, particularly internalizing problems and memory impairments given the integral role that the limbic structures play in memory, emotional processing and regulation^33^. This is particularly interesting given that, in comparison to males, females are more susceptible to persistent internalizing and anxiety problems in both normal and TBI populations^34–36^. Since these internalizing issues typically arise more chronically in response to injury, we may have revealed more exacerbated changes in glymphatic clearance rates indicative of inefficient waste clearance in the limbic structures if we examined MRI outcomes at longer-term time points.

The majority of the cortical structures examined (primary motor cortex, insular cortex, and DLPFC) did not show any significant differences between the RmTBI and sham injury groups, apart from a trend towards significance in the DLPFC. Contrary to our initial hypothesis, our non-significant results may actually also indicate an impairment in glymphatic system function. Our results for the cortical structures suggest that, for the most part, the RmTBI and sham animals exhibit no significant differences in glymphatic clearance rate. However, the RmTBI animals theoretically have more neurotoxic waste to clear from the CNS since TBI is associated with an increase in toxic metabolites and pro-inflammatory molecules^37–39^. Subsequently, a glymphatic clearance rate comparable to that of a sham animal may not be sufficient to accommodate this significant increase in waste that the RmTBI induces. This inefficient waste removal would then create an accumulation of neurotoxic waste within the CNS, which consequently produces the neurological deficits observed in post-concussive symptomology. Further investigation is required to determine if the lack of significant difference between RmTBI and sham glymphatic clearance rates is indicative of neurotoxic waste accumulation within the CNS.

The combination of an increased glymphatic influx and reduced efflux is likely to be maladaptive in numerous respects. For instance, the initial inflammatory cascade following mTBI performs numerous beneficial functions, including phagocytosis, isolation of healthy brain tissue, and the prevention of infection^40,41^. However, the accumulation of neuroinflammatory molecules, which is likely to occur as a consequence of this altered glymphatic flow, is a detrimental process that is commonly observed following TBI and has been linked to the associated post-injury deficits^37,39^. Cerebral edema, another common consequence of TBI^39^, may also be explained by these observed glymphatic flow changes since the increase in glymphatic influx likely delivers CSF throughout the brain at an elevated rate but the decrease in glymphatic efflux may not allow it to be adequately cleared. Additionally, an altered glymphatic flow rate may change the amount of glucose in the limbic regions post-injury, given that this system also functions to distribute compounds important for neurological health^3^. Patients with brain glucose levels above or below the normal range have been shown to exhibit poorer outcomes following TBI^40,42,43^. In this case, the combined increase in glymphatic influx and reduced efflux evident in the olfactory bulb and limbic structures could promote glucose toxicity in these regions. Moreover, the combination of increased brain glucose and glutamate levels following TBI has been shown to contribute to metabolic dysfunction and neuronal cell death, which correlates with worse outcomes^40,44^. Therefore, an increased glymphatic influx and decreased efflux likely disturb equilibriums of both glucose and the neuroinflammatory response, thereby altering the recovery process post-injury. However, these hypotheses are only speculative at this point, and further investigation is required to appropriately address these theories.

Given these hypotheses, it is likely that, like most biological systems, the glymphatic system is regulated by homeostatic mechanisms. When its clearance rate departs from its normal range, either above or below, its functionality likely becomes somewhat maladaptive. When the glymphatic clearance rate is above its normal range, it may clear away inflammatory molecules important for injury-related recovery and elevate brain glucose and glutamate levels, leading to dysfunction and cell death. When the glymphatic clearance rate is below its normal range or unresponsive to a changing neurological environment, it may be inefficient in removing macroscopic waste, especially following injury. Therefore, it seems that the glymphatic system may optimally function and promote neurological well-being within a particular range of flow rates.

Although AQP4 has been characterized as one of the key components of the glymphatic system, given that it is speculated to mediate the CSF-ISF exchange, more recent research has disputed these claims. The initial evidence in support of this characterization is derived from the investigation into the glymphatic clearance changes in response to AQP4 gene deletion. In this initial study, AQP4 null mice displayed a reduced glymphatic influx and clearance as well as suppression of *exogenous* amyloid-β clearance, indicating that this system was reliant on AQP4^2^. However, when a more recent study attempted to replicate these results, their results indicated that the AQP4 gene deletion mice demonstrated similar or slightly elevated dye penetration and amyloid-β distribution comparative to wildtype mice, suggesting that AQP4 did not influence glymphatic system function^45^. Given this controversy regarding AQP4’s contribution to the glymphatic system, we did not examine any AQP4 related measurements, although future studies should aim to elucidate these discrepancies.

Considering that the glymphatic system is 90% more active during sleep states^46^, it seems that a key function of sleep is to clear the brain of neurotoxic waste products that were produced during wakefulness^3^. This activity change during both natural sleep and anesthetized sleep is associated with a 60% expansion of the interstitial space, which drastically increases the glymphatic system’s convective CSF-ISF exchange^1^. Given that the glymphatic system is profoundly more active during sleep states, disturbed sleep is hypothesized to impede macroscopic waste removal, potentially increasing the accumulation of neurotoxic waste within the CNS and leading to impairments in neurological function. This is supported by the finding that as little as one night of sleep deprivation increases amyloid-β levels and promotes amyloid-β accumulation^47–49^. Interestingly, sleep-wake disturbances are commonly reported following TBI and have been associated with prolonged recovery and more severe post-concussive symptomology, especially in adolescents^50–52^. Consequently, it is likely that disturbed sleep is integral to the changes in glymphatic dynamics following TBI and, thus, should be investigated in relation to glymphatics and TBI in future studies.

There are several limitations to the current study that should be taken into consideration, especially for future studies examining the effects of RmTBI on glymphatic system function. First, for this study we used exclusively female rats, preventing us from making any conclusions as to whether sex differences exist in glymphatic system functionality following RmTBI. We frequently uncover sex differences in both behavioural and molecular analysis outcomes in other studies conducted in our laboratory^53,54^, thus sex differences likely exist in terms of the glymphatic system as well and should be considered in future studies. It is important to note, that we did not account for the rat’s estrous cycle as this study concluded before the rats reached puberty and typically begin cycling (~P50)^55,56^. Moreover, research shows that when rats are group housed, as they were in this study, their estrous cycles synchronize^57,58^. Therefore, if our rats were sexually mature and cycling, there would be limited variability in the phase of their estrous cycles within this study. Second, we restricted our MRI analysis to one time point, one-day post-injury, yet it is possible that we missed another critical time point before or after this. Future research should examine a variety of different time points post-injury to develop a more detailed timeline of how RmTBI alters glymphatic clearance rate. Third, since the contrast-enhanced MRI procedure occurred at one day post-injury, behavioural data collection post-injury was limited to one behavioural test, the beam walking task. Future studies should expand the behavioural test battery to include analysis of other common post-injury impairments, including cognitive and emotional deficits. Finally, all behavioural testing and MR imaging took place during daylight hours, which is the rat’s sleep phase given that they are nocturnal animals. It is possible that the results of both the behavioural tests and MR imaging would be significantly different if we conducted them during the rat’s active phase, especially given that the glymphatic system’s activity is altered so drastically with the sleep/wake cycle.

Overall, this study suggests that the adolescent glymphatic system responds to RmTBI in a region-specific manner, wherein the clearance rate throughout the olfactory bulb and limbic structures is reduced but is unaltered in the cortical structures examined. This finding may indicate a role for an impaired and/or inefficient glymphatic system in the limbic structures and cortical structures, respectively, in the development of post-mTBI symptomology. However, future investigation into brain region-specific quantification of macroscopic waste, including amyloid-β and tau, as well as the inflammatory response and glucose levels in these animals is required to justify this claim. Furthermore, it is important to consider that adolescence represents a unique developmental period in which dramatic changes to brain structure and function are taking place^59,60^, making their outcomes distinct from those of any other developmental stage. Importantly, this study is the first, to the best of our knowledge, to examine the glymphatic system in adolescence, which reflects an important but unexplored field of research. Thus, this study poses a novel emphasis on the potential role of the glymphatic system in the development of post-concussive symptomology following RmTBI in adolescence.
